# Maternal Blood-Based Protein Biomarkers in Relation to Abdominal Fat Distribution Measured by Ultrasound in Early Mid-Pregnancy

**DOI:** 10.1007/s43032-022-00876-4

**Published:** 2022-02-11

**Authors:** Emelie Lindberger, Anna-Karin Wikström, Inger Sundström Poromaa, Fredrik Ahlsson

**Affiliations:** Department of Women’s and Children’s Health, Uppsala University, 751 85 Uppsala, Sweden

**Keywords:** Biomarkers, Body fat distribution, Pregnancy, Ultrasound

## Abstract

The objective of this study was to examine the associations of early mid-pregnancy ultrasound measured visceral and subcutaneous fat depths with blood-based protein biomarkers. This was a cross-sectional study including 201 pregnant women at Uppsala University Hospital, Sweden. The mean age of the women was 31.0 years, and 57.7% were nulliparous. Maternal visceral and subcutaneous fat depths were measured by ultrasound at the early second-trimester anomaly scan. A non-fasting blood sample was collected in conjunction with the second-trimester anomaly scan, and the Olink cardiovascular II panel was used to measure 92 blood-based protein biomarkers in the sample. Cross-sectional associations of visceral and subcutaneous fat depths with blood-based protein biomarkers were examined using Mann–Whitney *U* tests with false discovery rate adjustments. In addition, linear regression analyses adjusting for maternal age, parity, and early pregnancy body mass index were performed. The results showed differences in one biomarker between women with elevated (≥ 52 mm) versus normal (< 52 mm) visceral fat depth, and in three biomarkers between women with elevated (≥ 22 mm) versus normal (< 22 mm) subcutaneous fat depth. Hence, levels of blood-based protein biomarkers differ between pregnant women with dissimilar body fat distributions, which might reflect disparities in biological pathways.

**Supplementary Information:**

The online version contains supplementary material available at 10.1007/s43032-022-00876-4.

## Introduction

The global burden of overweight and obesity continues unabated [1]. Around 20% of pregnant women globally are overweight and 10% are obese [2]. Excessive body weight is a major cause of maternal and infant morbidity [3–6].

Fat tissue is not solely a depot for energy storage; it has endocrine properties as well [7]. It produces adipokines that are involved in appetite regulation, metabolism of nutrients, insulin sensitivity, and inflammation [8]. The adipokine production varies depending on the amount of fat tissue. In general, obese individuals have overproduction of pro-inflammatory adipokines and lower levels of adipokines that are anti-inflammatory and that promote insulin sensitivity compared with normal weight subjects [9]. It is suggested that the dysregulated adipokine secretion is contributing to the development of obesity-related complications [9].

There is a growing interest in identification and measurement of blood-based biomarkers related to obesity and its complications [10]. Biomarkers have the potential to give new information on pathophysiological pathways and could possibly be used in addition to anthropometry measures to characterize obesity phenotypes [10]. However, despite the growing number of studies evaluating biomarkers in relation to obesity, the significance of many of them is still unclear [10].

In non-pregnant individuals, body fat distribution is associated with risk of obesity-related complications [11]. Excessive fat stored within the abdominal cavity (i.e., visceral fat) is highly associated with complications such as cardiovascular disease, type 2 diabetes, and different cancer forms [11, 12]. The causal pathways are not fully understood, but insulin resistance and low-grade inflammation are proposed as possible mechanisms [11, 13, 14].

Whether body fat distribution affects the risk for pregnancy complications is not fully elucidated. We have previously reported on independent associations of maternal visceral fat depth in early mid-pregnancy with infant birth size [15]. Our results indicate differences in factors involved in fetal development between women with different body fat distributions. Differences in blood-based biomarkers between women with predominantly visceral fat accumulation in comparison with abdominal subcutaneous fat could possibly reflect metabolic changes induced by these fat tissue compartments, such as insulin sensitivity and inflammation. Pregnancy is characterized by mild maternal systemic inflammation mediated by immunomodulatory hormones and cytokines from the placenta [16]; hence, an inflammatory response could be mediated by fat distribution or by the pregnancy itself.

To the best of our knowledge, protein biomarker profiles in relation to fat distribution in pregnant women have not previously been studied. We hypothesize that levels of blood-based protein biomarkers differ between pregnant women with different fat distributions and that these biomarkers could clarify cause and mechanisms of the underlying biological processes behind an association of fat distribution with pregnancy outcomes. With this study, we sought to describe the observational associations of ultrasound measured fat distribution in early mid-pregnancy with 92 blood-based protein biomarkers in a cohort of 201 women.

## Material and Methods

Data for this cross-sectional study were derived from two studies at the Department of Women’s and Children’s Health, Uppsala University Hospital. Both studies were approved by the Regional Ethical Review Board in Uppsala (Dnr: 2014/353, Dnr: 2019–00391, and Dnr: 2007/181). All research was performed in accordance with relevant national and international guidelines.

Information on visceral fat depth (VF) and subcutaneous fat depth (SCF) was derived from a cohort study at Uppsala University Hospital conducted between January 2015 and January 2019. Ethical approval was obtained to implement a new clinical routine, VF and SCF measurements, and to evaluate this routine by linkage to standardized hospital electronic medical records on maternal, obstetric, and perinatal health care. Informed consent was waived by the Swedish Ethical Review Authority (Dnr: 2019–00391). Eligible study participants were women attending a second-trimester anomaly scan at this hospital from January 2015 to January 2019. During this period, 4039 women underwent a second-trimester anomaly scan that included fat depth measurements. This corresponds to approximately 25% of the total number of women undergoing a second-trimester anomaly scan during the study period. It was a matter of coincidence if the scan was performed by a midwife trained in fat depth measurements, since the personnel booking ultrasonography appointments were not involved in the study. The fat depth measures were taken as per Armellini et al. [17], with a minor modification of the placement of the probe. The measuring point was located at the body’s midline 10 cm above the umbilicus. The VF was defined as the distance in millimeters from the inner border of the rectus abdominis muscle to the anterior border of the aorta. The SCF was defined as the distance in millimeters from the dermis to the surface of the rectus abdominis muscle. The fat depth measures were assessed using a GE Voluson E6, E8, or E10 ultrasound machine (GE Medical Systems, Zipf, Austria). All midwives that performed the measurements were certified obstetric ultra-sonographers. During the study period, additional training sessions were carried out in order to maximize the quality of the scans. Moreover, the intraclass correlation coefficient of the inter-examiner variation was 0.83 for VF measures, and 0.85 for SCF measures, indicating good reliability [18].

The following information was extracted from the women’s standardized antenatal electronic medical records: body mass index (BMI) (kg/m^2^), age (years), parity (nulliparous or parous), and maternal country of birth (EU or outside EU). Information on chronic illnesses was filled in by the midwife at the first antenatal visit using checkboxes in the standardized antenatal electronic medical record. Data were also obtained from the women’s standardized antenatal electronic medical records on the following diagnoses according to the International Classification of Diseases 10 (ICD-10): diabetes mellitus type 1 and type 2 (E10, E11), rheumatic disease (L40, M05, M32, M35, M45), epilepsy (G40), inflammatory disease (D69, K50, K51, K90), essential hypertension (I10), and endocrine disease (E03).

Blood samples were collected as part of the population-based Uppsala Biobank for Pregnant Women, where blood samples are collected in conjunction with the second-trimester anomaly scan since 2007. Eligible women are 18 years or older, Swedish-speaking, and without blood-borne disease (HIV, hepatitis C, and hepatitis B). Invitation to participate in the Biobank is done at random, when a research nurse is available. Approximately 30% of the respondents decline participation, and the Biobank covers approximately half of the pregnant population of Uppsala County [19]. Following written informed consent, a blood sample is collected. The sample is centrifuged within two hours and stored at − 70 °C.

By June 2019, 202 women in the VF and SCF measurement cohort had donated a blood sample in the Uppsala Biobank for Pregnant Women. One individual was excluded from further analysis due to blood sample analytical fault. Hence, the final cohort consisted of 201 pregnant women. Following linkage, the study population database was anonymized.

### Proteomics Assay

The Olink cardiovascular II panel measures 92 protein biomarkers either known to be or suspected to be markers of inflammatory and cardiovascular disease in humans. It is based on a proximity assay technology developed at the Clinical Biomarkers Facility, Science for Life Laboratory, Uppsala. The Olink proximity extension assay measures individual protein profiles. Pairs of antibodies marked with unique DNA tags bind to the protein in the sample. When two matched DNA tags come in close proximity, they bind to each other. The hybridized DNA tags are extended to an amplicon and a unique code is generated for each protein. Next, qPCR is used to read out the protein profile. The number of qPCR cycles is used to calculate the protein concentration, and the relative concentration is reported. The results are given as Normalized Protein eXpression (NPX) values, an arbitrary unit in log2 scale where a high protein value corresponds to a high protein concentration [20, 21]. Full names of the protein biomarkers included in the Olink cardiovascular II panel are presented in Supplementary Table 1 (Online Resource).

### Protein Interaction Analysis

In order to detect possible interactions between the proteins that were different between groups, the STRING database for protein–protein interaction networks functional enrichment analysis online (http://string-db.org/) was used [22]. The minimum required interaction score was set at 0.4. The interaction score is a confidence indicator and 0.4 implies medium level of confidence [23].

### Statistical Analyses

All statistical analyses were performed using IBM SPSS Statistics version 27. Visceral fat depth and SCF were categorized in quartiles (VF quartiles 1‒4 and SCF quartiles 1‒4). A threshold point was set at quartile 4 (VF ≥ 52 mm and SCF ≥ 22 mm, referred to as “elevated”), and quartiles 1‒3 constituted the reference group (referred to as “normal”).

The outcome data were not normally distributed. Non-parametric Mann–Whitney *U* tests adjusting for multiple testing (false discovery rate) were used to identify biomarkers that were different between groups (quartiles 1‒3 vs. quartile 4). Additionally, we performed multiple linear regression analyses adjusting for maternal age, parity, and early pregnancy BMI to correct for potential confounding factors. Only biomarkers that differed between groups were analyzed. A nominal two-sided *P*-value < 0.05 was considered indicating statistical significance.

## Results

The women had a mean age of 31.0 years (range 20‒45 years), 116 (57.7%) were nulliparous, and 72 (35.8%) had overweight or obesity. Information on BMI was missing in one individual. The clinical characteristics are described in Table 1.

**Table 1:** Descriptive characteristics of the study population

	Variable	Cohort
Women	*N*	201
	Age, years (mean, range)	31.0 (20‒45)
	Nulliparous, *n* (%)	116 (57.7)
	Early pregnancy BMI kg/m^2^ (mean ± SD)	25.3 ± 5.2
	BMI < 18.5 kg/m^2^ (underweight), *n* (%)	3 (1.5)
	BMI 18.5‒24.9 kg/m^2^ (normal weight), *n* (%)	125 (62.5)
	BMI 25.0‒29.9 kg/m^2^ (overweight), *n* (%)	38 (19.0)
	BMI ≥ 30.0 kg/m^2^ (obesity), *n* (%)	34 (17.0)
	Country of birth within the EU, *n* (%)	186 (92.5)
	Diabetes mellitus type 1 or type 2, *n* (%)	0 (0.0)
	Rheumatic disease, *n* (%)	1 (0.5)
	Epilepsy, *n* (%)	0 (0.0)
	Inflammatory disease, *n* (%)	3 (1.5)
	Essential hypertension, *n* (%)	1 (0.5)
	Endocrine disease^a^, *n* (%)	11 (5.5)

*BMI* body mass index, *SD* standard deviation

^a^Hypothyroidism, thyrotoxicosis

### *VF and SCF Measures in Relation to Biomarker Levels*

The VF and SCF measurements were performed at mean gestational age 133 days (standard deviation ± 5.2 days). The VF ranged from 9 to 83 mm, and SCF from 4 to 46 mm.

The following biomarkers were excluded from analysis due to a substantial proportion of the women having values below the limit of detection: ITGB1BP2 (melusin) (48.8% below level of detection), BNP (natriuretic peptides B) (43.3% below level of detection), and CA5A (carbonic anhydrase 5A, mitochondrial) (37.3% below level of detection), leaving 89 protein biomarkers for analysis.

**Fig. 1:**
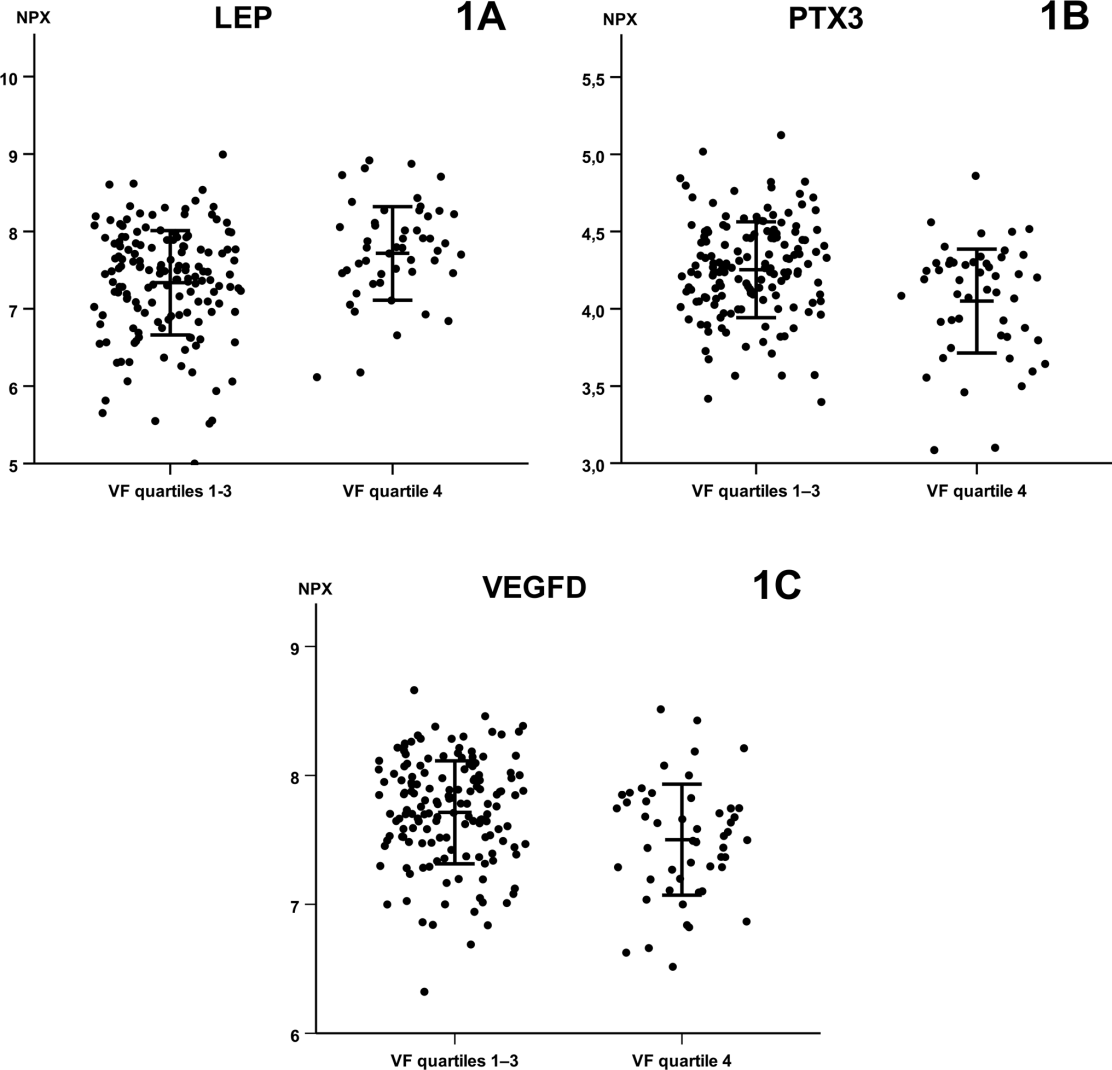
Individual levels of three blood-based protein biomarkers differing between pregnant women with normal and elevated visceral fat depth. Data are given as normalized protein expression (NPX) log2. Data were analyzed using Mann–Whitney U tests with false discovery rate (FDR) adjustments. Horizontal bars represent mean ± SD. *P* < 0.001 for LEP and PTX3, *P* = 0.001 for VEGFD. LEP, leptin; PTX3, pentraxin-related protein PTX3; VEGFD, vascular endothelial growth factor D

Three biomarkers differed between women with elevated versus normal VF in the unadjusted analysis (Fig. 1). One biomarker was higher (LEP [leptin]) in women with elevated VF (Fig. 1 panel A), and two biomarkers were lower (PTX3 [pentraxin-related protein PTX3] (Fig. 1 panel B), VEGFD [vascular endothelial growth factor D]) (Fig. 1 panel C) compared with women with normal VF.

**Fig. 2:**
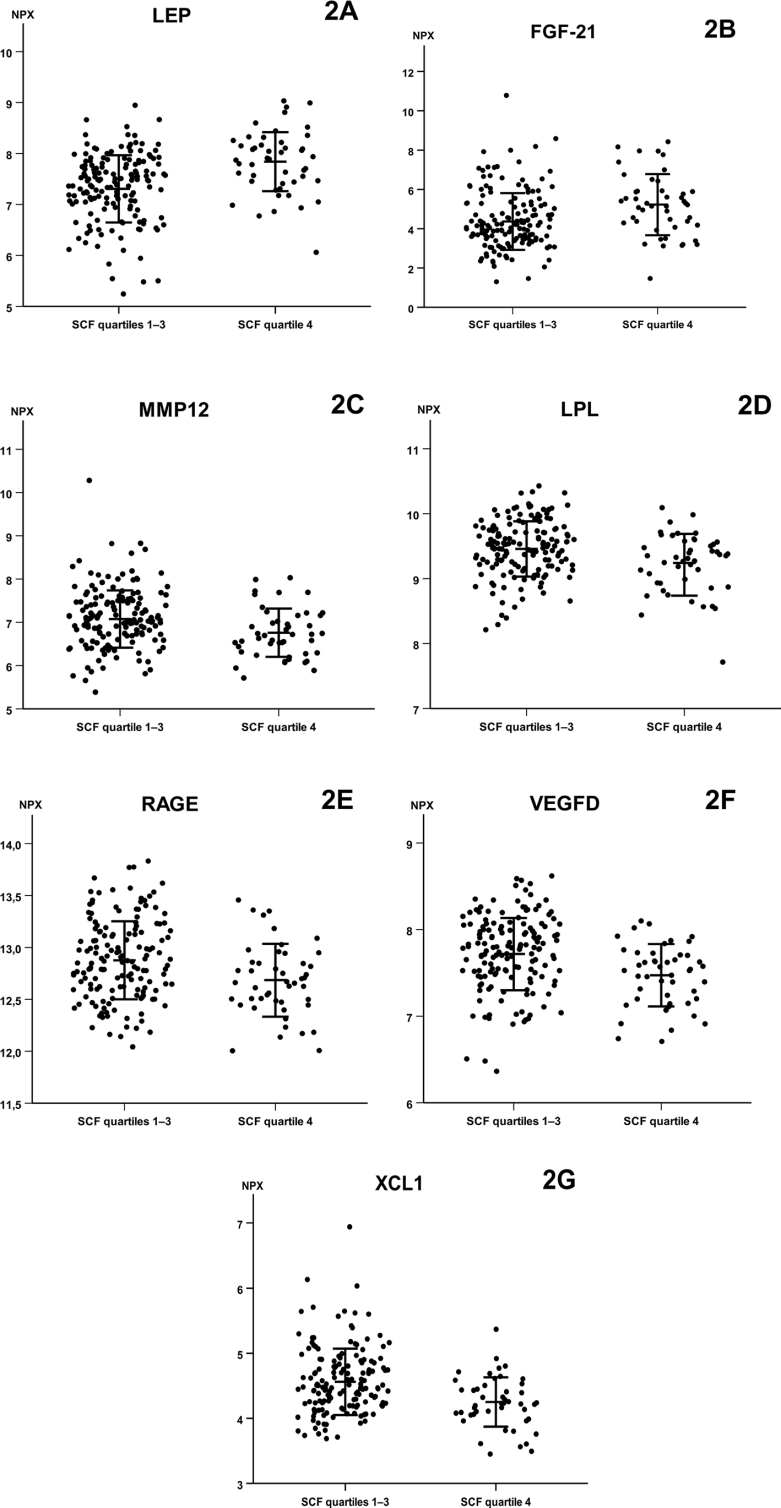
Individual levels of seven blood-based protein biomarkers differing between pregnant women with normal and elevated subcutaneous fat depth. Data are given as normalized protein expression (NPX) log2. Data were analyzed using Mann–Whitney *U* tests with false discovery rate (FDR) adjustments. Horizontal bars represent mean ± SD. *P* < 0.001 for FGF-21, LEP, VEGFD, and XCL1, *P* = 0.003 for LPL, MMP12, and RAGE. FGF-21, fibroblast growth factor 21; LEP, leptin; LPL, lipoprotein lipase; MMP12, matrix metalloproteinase-12; RAGE, receptor for advanced glycosylation end products; VEGFD, vascular endothelial growth factor D; XCL1, lymphotactin

Seven biomarkers differed between women with elevated versus normal SCF in the unadjusted analysis (Fig. 2). In women with elevated SCF, two biomarkers were higher (LEP [leptin] (Fig. 2 panel A), FGF-21 [fibroblast growth factor 21]) (Fig. 2 panel B) and five biomarkers were lower (MMP-12 [matrix metalloproteinase-12] (Fig. 2 panel C), LPL [Lipoprotein lipase] (Fig. 2 panel D), RAGE [receptor for advanced glycosylation end products] (Fig. 2 panel E), VEGFD [vascular endothelial growth factor D] (Fig. 2 panel F), and XCL1 [lymphotactin]) (Fig. 2 panel G) compared with women with normal SCF.

To correct for potential confounding factors, multiple linear regression analyses adjusting for maternal age, parity, and early pregnancy BMI were performed. After adjustments, we found that one biomarker (PTX3) remained different between women with elevated versus normal VF (B coefficient (*β*) − 0.18, 95% confidence interval (CI) − 0.26 to − 0.02) (Table 2). In addition, three biomarkers remained different between women with elevated versus normal SCF (FGF-21 (*β* 0.19, CI 0.03 to 1.27), LPL (*β* − 0.21, CI − 0.40 to − 0.03), and XCL1 (*β* − 0.26, CI − 0.51 to − 0.10)).

**Table 2:** Associations between body fat distribution and blood-based biomarker levels

Body fat distribution	Biomarker	Adjusted model^a^		
		*β*	CI	*P*
Elevated (≥ 52 mm) vs. normal (< 52 mm) visceral fat depth	LEP	0.00	− 0.23 to 0.23	0.997
	PTX3	− **0.18**	− **0.26 to − 0.02**	**0.026**
	VEGFD	− 0.08	− 0.23 to 0.07	0.288
Elevated (≥ 22 mm) vs. normal (< 22 mm) subcutaneous fat depth	FGF-21	**0.19**	**0.03 to 1.27**	**0.039**
	LEP	0.07	− 0.14 to 0.37	0.391
	LPL	− **0.21**	− **0.40 to** − **0.03**	**0.022**
	MMP-12	− 0.13	− 0.47 to 0.07	0.154
	RAGE	− 0.07	− 0.21 to 0.10	0.453
	VEGFD	− 0.09	− 0.25 to 0.08	0.308
	XCL1	− **0.26**	− **0.51 to** − **0.10**	**0.004**

Data are *B* coefficients (*β*) and 95% confidence intervals (CI) for the change in outcome depending on body fat distribution. Significant results are in bold

*FGF-21* fibroblast growth factor 21, *LEP* leptin, *LPL* lipoprotein lipase, *MMP-12* matrix metalloproteinase-12, *PTX3* pentraxin-related protein PTX3, *RAGE* receptor for advanced glycosylation end products, *VEGFD* vascular endothelial growth factor D, *XCL1* lymphotactin

^a^Adjustments were made for maternal age, parity, and early pregnancy BMI

#### Protein Interaction Analysis

The interaction analysis did not show any interactions between the proteins that were different between groups (Supplementary Fig. 1, Online Resource). However, the automated text mining performed by the STRING database showed that two of the proteins (FGF-21 and LPL) co-occur in PubMed abstracts. Of note, the text mining performed by the STRING database does not relate the proteins to the context of this study.

## Discussion

We found differences in the levels of four blood-based protein biomarkers between pregnant women with dissimilar body fat distributions. Among 92 blood-based protein biomarkers either known to be or suspected to be markers of inflammatory and cardiovascular disease, one biomarker was different between women with elevated versus normal VF, and three biomarkers were different between women with elevated versus normal SCF after adjustments for maternal age, parity, and early pregnancy BMI.

We found that the levels of PTX3 were lower in pregnant women with elevated VF. PTX3 is involved in innate immune responses, inflammatory reactions, and female fertility. It belongs to the same family as C-reactive protein (CRP) and functions as a soluble pattern recognition receptor [24]. It also plays a role in female fertility by organizing the extracellular matrix of the cumulus oophorus [24, 25]. PTX3 is suggested as a biomarker of oocyte quality [26]. During normal pregnancy, circulating PTX3 levels increase compared with the pre-pregnancy state, but do not change between the trimesters [27]. The levels of PTX3 are further elevated in individuals with pre-eclampsia compared with women with normal pregnancies [27]. The authors hypothesize that the elevated PTX3 levels seen in pregnancies complicated by pre-eclampsia could be a marker of impaired function of the endothelium. In addition, PTX3 is proposed to be involved in metabolic control. A study investigating a cohort consisting of 27 normal weight and 48 overweight men reports that PTX3 levels are inversely correlated with triglyceride levels during fasting. Additionally, the authors report an inverse correlation between PTX3 levels and insulin secretion as well as glucose concentration after oral and intravenous administration of glucose [28]. These findings are in line with our results showing lower levels of PTX3 in pregnant women with an elevated VF measure, because VF accumulation is associated with insulin resistance and glycemia in both non-pregnant and pregnant individuals [11, 29]. Moreover, the study also reports on plasma PTX3 levels in another cohort consisting of 19 normal weight, 28 overweight, and 15 obese individuals. The results show that plasma PTX3 levels are inversely associated with body weight and waist-to-hip ratio [28], findings that are confirming previous research [30], and are consistent with our results.

In our analysis, FGF-21 levels were higher in pregnant women with elevated SCF. FGF-21 activates glucose uptake in adipocytes [31] and is suggested to prevent the development of diabetes mellitus and obesity by its ability to normalize glucose and lipid homeostasis [32]. However, elevated levels of FGF-21 are reported in individuals with type 2 diabetes, obesity, and cardiovascular disease, and FGF-21 is therefore suggested as a biomarker of these conditions [32, 33]. It is suggested that the increased levels of FGF-21 in individuals with obesity-related metabolic dysfunction could be a physiologic response to counterbalance metabolic stress. Another proposed explanation is that obesity leads to FGF-21 resistance and that FGF-21 levels are increased in obese subjects as a compensatory mechanism [33]. In pregnant women, FGF-21 levels are positively correlated with BMI and adiposity [34]. There is an increase in FGF-21 levels from the first to the third trimester, but no association of changes in FGF-21 levels with pregnancy weight gain. Furthermore, there is an inverse relation between FGF-21 levels and maternal glucose concentration. The authors outlining the above findings speculate that FGF-21 levels seem unresponsive to changes in maternal energy depots but might reflect maternal macronutrient status [34]. We found that pregnant women with elevated SCF had higher levels of FGF-21, whereas a study investigating FGF-21 levels in relation to abdominal subcutaneous fat measured by magnetic resonance imaging reports no association [35]. The contradictive results could possibly be explained by differences in study population characteristics, we investigated pregnant women while the other study only included adolescent subjects. As far as we know, FGF-21 in relation to VF and SCF measures during pregnancy have not previously been investigated.

Our results showed that LPL was lower in pregnant women with elevated SCF. LPL is an enzyme that hydrolyzes the triacylglycerol component in circulating lipoproteins, such as chylomicrons and very low density lipoproteins (VLDL). LPL is also involved in the cellular uptake of components from chylomicrons, cholesterol-containing lipoproteins, and free fatty acids [36]. LPL is produced by many tissues and cells, such as fat tissue, heart, muscle, white blood cells [37], and placenta [38]. The activity of LPL is responsive to nutritional status and hormonal changes [37]. In early pregnancy, the LPL activity in fat tissue increases [39], promoting lipid accumulation in maternal fat stores [40]. Later in pregnancy, the fat tissue LPL activity decreases [41], contributing to the breakdown of maternal fat depots [40]. LPL mass in human preheparin serum is suggested as a biomarker of obesity, insulin resistance, and dyslipidemia, and LPL mass is inversely related to the metabolic syndrome [37, 42]. Our findings, that women with elevated SCF had lower LPL levels, are in good agreement with the results from a previous study reporting a negative correlation between second-trimester LPL mass and maternal abdominal subcutaneous fat tissue in early pregnancy [43]. The authors hypothesize that low LPL levels might indicate a decreased synthesis of LPL by insulin-resistant adipocytes in the abdominal subcutaneous fat tissue.

Lastly, we found lower XCL1 levels in pregnant women with elevated SCF. XCL1 is a cytokine that belongs to the XC chemokine family. The function of XCL1 is chemotactic activation of lymphocytes, and it is thereby involved in inflammatory and immunological responses [44]. Interestingly, XCL1 levels are down-regulated in the receptive endometrium during the window of implantation in obese patients [45]. The specific role of XCL1 in implantation is unknown. However, the authors outlining the above findings suggest that altered gene expression in the endometrium might be a result of metabolic dysfunction related to obesity, and that altered gene expression could contribute to decreased implantation rates and increased rates of miscarriage reported in obese patients with infertility [45]. Our finding, that pregnant women with elevated SCF had lower XCL1 levels, has not been reported earlier. As far as we know, the relation between XCL1 and body fat distribution has not previously been investigated. Further research is needed to elucidate the relation between low XCL1 levels and increased SCF during pregnancy.

The strengths of this study include a large sample size, and a wide range of blood-based protein biomarkers included in the analyses. Ultrasound assessment of intra-abdominal fat tissue has been evaluated concerning validity and reproducibility, and strong correlations between ultrasound and CT scan measurements (*r* = 0.81; *P* < 0.001) are reported [46]. A possible limitation is that the Olink biomarkers are reported as relative values and not absolute values, which could complicate comparisons with other studies. However, a study investigating preeclampsia subtypes using an Olink CVD biomarker panel compares the result of one biomarker (PlGF) with that from an immunochemiluminescence assay and reports an excellent correlation [47]. Another limitation is that the Olink protein biomarkers were measured only once. Biomarker levels could vary within one individual over time [10]. However, the Olink panels are expensive, and it might not have been realistic from an economic point of view to perform additional analyses. Furthermore, a biomarker might be involved in other biological pathways besides the one studied, which might be difficult to account for [10]. The placenta also contributes to the production of blood-based protein biomarkers and associations may therefore not only be linked to the fat tissue. Lastly, other tests might reflect insulin sensitivity better than the proteins included in the Olink biomarker panel that was used in this study. If a similar study would be conducted in the future, it would be preferable to include for example HbA1c or perform oral glucose tolerance tests to obtain a better measure of insulin sensitivity.

As stated by Herrera et al. [48], a better understanding for biological processes linking maternal obesity with adverse perinatal outcomes is needed. At present time, no biomarkers are fulfilling the criteria to be used in clinic to predict perinatal complications [48]. Although previous studies support theories of inflammatory pathways, actions of specific inflammatory mediators, and inflammatory properties of the placenta, much is unknown and more research is needed [48].

## Conclusion

Pregnant women with disparate body fat distributions have different levels of blood-based protein biomarkers related to inflammation as well as lipid and glucose metabolism. The differences in biomarker levels might reflect disparities in biological pathways related to inflammatory and metabolic processes. Further exploration of blood-based biomarkers during pregnancy could possibly elucidate biological patterns linking body fat distribution types to perinatal outcomes.

## Supplementary Information

Below is the link to the electronic supplementary material.

Supplementary file1 (PDF 361 KB)

## Data Availability

The datasets generated during and/or analyzed during the current study are available from the corresponding author on reasonable request.
