# Supplementary material for: Maternal Blood-Based Protein Biomarkers in Relation to Abdominal Fat Distribution Measured by Ultrasound in Early Mid-Pregnancy
Source: Reprod Sci. 2022 Feb 11;29(8):2333–41. doi: 10.1007/s43032-022-00876-4 (PMC9352629; doi:10.1007/s43032-022-00876-4)
Supplement: Supplementary file 1 — Supplementary file1 (PDF 361 KB) [file 43032_2022_876_MOESM1_ESM.pdf]

Supplementary information file

Maternal blood-based protein biomarkers in relation to abdominal fat distribution measured by ultrasound in early mid pregnancy

Reproductive Sciences

Emelie Lindberger MD PhD[1*], Anna-Karin Wikström MD PhD[1], Inger Sundström Poromaa MD PhD[1], Fredrik Ahlsson MD PhD[1]

[1] Department of Women's and Children's Health, Uppsala University, 751 85 Uppsala, Sweden

Corresponding author

Emelie Lindberger
Department of Women's and Children's Health, Uppsala University, 751 85 Uppsala, Sweden
0046706346751, emelie.lindberger@kbh.uu.se

Supplementary Table 1. List of the 92 protein biomarkers included in the Olink Cardiovacular II panel

Abbreviation	Protein name
DECR1	2,4-dienoyl-CoA reductase, mitochondrial
ADAM-TS13	A disintegrin and metalloproteinase with thrombospondin motifs 13
ADM	ADM
AGRP	Agouti-related protein
IDUA	Alpha-L-iduronidase
ANGPT1	Angiopoietin-1
TIE2	Angiopoietin-1 receptor
ACE2	Angiotensin-converting enzyme 2
BMP-6	Bone morphogenetic protein 6
BOC	Brother of CDO
CA5A	Carbonic anhydrase 5A, mitochondrial
CEACAM8	Carcinoembryonic antigenrelated cell adhesion molecule 8
CTSL1	Cathepsin L1
CCL17	C-C motif chemokine 17
CCL3	C-C motif chemokine 3
CD40-L	CD40 ligand
CTRC	Chymotrypsin C
CXCL1	C-X-C motif chemokine 1
DCN	Decorin
Dkk-1	Dickkopf-related protein 1
FABP2	Fatty acid-binding protein, intestinal
FGF-21	Fibroblast growth factor 21
FGF-23	Fibroblast growth factor 23
FS	Follistatin
Gal-9	Galectin-9
GIF	Gastric intrinsic factor
GT	Gastrotropin
GH	Growth hormone
GDF-2	Growth/differentiation factor 2
HSP 27	Heat shock 27 kDa protein
HO-1	Heme oxygenase 1
HAOX1	Hydroxyacid oxidase 1
IL-1ra	Interleukin-1 receptor antagonist protein
IL1RL2	Interleukin-1 receptor-like 2
IL-17D	Interleukin-17D
IL-18	Interleukin-18
IL-27	Interleukin-27
IL-4RA	Interleukin-4 receptor subunit alpha
IL-6	Interleukin-6
KIM1	Kidney injury molecule 1
GLO1	Lactoylglutathione lyase
LOX-1	Lectin-like oxidized LDL receptor 1
LEP	Leptin

LPL	Lipoprotein lipase
IgG Fc receptor II-b	Low affinity immunoglobulin gamma Fc region receptor II-b
XCL1	Lymphotactin
MARCO	Macrophage receptor MARCO
MMP12	Matrix metalloproteinase-12
MMP7	Matrix metalloproteinase-7
ITGB1BP2	Melusin
BNP	Natriuretic peptides B
NEMO	NF-kappa-B essential modulator
hOSCAR	Osteoclast-associated immunoglobulinlike receptor
PAPPA	Pappalysin-1
PTX3	Pentraxin-related protein PTX3
PGF	Placenta growth factor
PDGF subunit B	Platelet-derived growth factor subunit B
PARP-1	Poly [ADP-ribose] polymerase 1
PIgR	Polymeric immunoglobulin receptor
PD-L2	Programmed cell death 1 ligand 2
HB-EGF	Proheparin-binding EGF-like growth factor
IL16	Pro-interleukin-16
PRELP	Prolargin
PRSS8	Prostasin
AMBP	Protein AMBP
PAR-1	Proteinase-activated receptor 1
TGM2	Protein-glutamine gammaglutamyltransferase 2
SRC	Proto-oncogene tyrosine-protein kinase Src
PSGL-1	P-selectin glycoprotein ligand 1
RAGE	Receptor for advanced glycosylation end products
REN	Renin
PRSS27	Serine protease 27
STK4	Serine/threonine-protein kinase 4
SERPINA12	Serpin A12
CD84	SLAM family member 5
SLAMF7	SLAM family member 7
SORT1	Sortilin
SPON2	Spondin-2
SCF	Stem cell factor
SOD2	Superoxide dismutase [Mn], mitochondrial
CD4	T-cell surface glycoprotein CD4
TM	Thrombomodulin
THPO	Thrombopoietin
THBS2	Thrombospondin-2
TF	Tissue factor
TRAIL-R2	TNF-related apoptosis-inducing ligand receptor 2
TNFRSF10A	Tumor necrosis factor receptor superfamily member 10A
TNFRSF11A	Tumor necrosis factor receptor superfamily member 11A
TNFRSF13B	Tumor necrosis factor receptor superfamily member 13B
MERTK	Tyrosine-protein kinase Mer

VEGFD	Vascular endothelial growth factor D
VSIG2	V-set and immunoglobulin domaincontaining protein 2

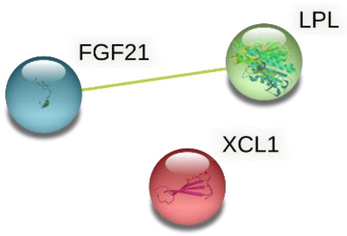

Supplementary Fig. 1 Protein network analysis (using the STRING database version 11.0) of biomarkers differing between normal and elevated subcutaneous fat depth showed that FGF-21 and LPL are mentioned together in PubMed abstracts (indicated by yellow line). No interactions were found. FGF21, Fibroblast growth factor 21; LPL, Lipoprotein lipase; XCL1, Lymphotactin
